# DACL-Net: A Dual-Branch Attention-Based CNN-LSTM Network for DOA Estimation

**DOI:** 10.3390/s26020743

**Published:** 2026-01-22

**Authors:** Wenjie Xu, Shichao Yi

**Affiliations:** 1 School of Computer, Jiangsu University of Science and Technology, Zhenjiang 212003, China; 231210701126@stu.just.edu.cn; 2 School of Science, Jiangsu University of Science and Technology, Zhenjiang 212003, China; 3 Zhenjiang Jizhi Ship Technology Co., Ltd., Zhenjiang 212003, China

**Keywords:** DOA estimation, acoustic vector array, signal processing, attention, deep learning

## Abstract

While deep learning methods are increasingly applied in the field of DOA estimation, existing approaches generally feed the real and imaginary parts of the covariance matrix directly into neural networks without optimizing the input features, which prevents classical attention mechanisms from improving accuracy. This paper proposes a spatio-temporal fusion model named DACL-Net for DOA estimation. The spatial branch applies a two-dimensional Fourier transform (2D-FT) to the covariance matrix, causing angles to appear as peaks in the magnitude spectrum. This operation transforms the original covariance matrix into a dark image with bright spots, enabling the convolutional neural network (CNN) to focus on the bright-spot components via an attention module. Additionally, a spectrum attention mechanism (SAM) is introduced to enhance the extraction of temporal features in the time branch. The model learns simultaneously from two data branches and finally outputs DOA results through a linear layer. Simulation results demonstrate that DACL-Net outperforms existing algorithms in terms of accuracy, achieving an RMSE of 0.04° at an SNR of 0 dB.

## 1. Introduction

Direction-of-arrival (DOA) estimation is a cornerstone of array signal processing, with extensive applications in radar, wireless communications, electronic countermeasures, acoustic direction finding, and astronomy. It aims to precisely determine the incident angles of signals impinging on an antenna array, which is an indispensable prerequisite for subsequent operations such as target localization and tracking [1,2,3,4]. DOA estimation plays a pivotal role in state-of-the-art systems, including MIMO communications, intelligent transportation networks, UAV collaborative operations, and 6G infrastructure [5,6,7]. For example, in MIMO systems, it enables enhanced beamforming and optimized spatial multiplexing [8,9,10], while in radar, it strengthens the ability to detect and track multiple targets simultaneously. Consequently, the development of DOA estimation methods that achieve high precision and low computational complexity, particularly under adverse operating conditions, remains a central focus of academic and industrial research [11,12].

Over decades of research, a wealth of classical DOA estimation approaches have been devised. Conventional beamforming (CBF), one of the earliest developed techniques, estimates signal directions through the construction of spatial beam patterns [13,14,15]. Nevertheless, its effectiveness is constrained by the Rayleigh resolution criterion and vulnerability to noise when operating in low signal-to-noise ratio (SNR) scenarios [16,17,18]. To address these drawbacks, super-resolution subspace-based algorithms were proposed [19,20,21,22,23,24,25], including multiple signal classification (MUSIC) [21,22,23] and estimation of signal parameters via rotational invariance techniques (ESPRIT) [24,25]. MUSIC capitalizes on the orthogonality between signal and noise subspaces, but it entails substantial computational costs and exhibits inferior performance when dealing with coherent sources. In contrast, ESPRIT enhances efficiency by utilizing the rotational invariance property across subarrays, though it still encounters constraints under specific operating conditions. Additional methods, such as minimum variance distortionless response (MVDR) and maximum likelihood (ML) estimation, provide higher accuracy or theoretical optimality. However, they are frequently deemed impractical due to their heavy computational requirements, or sensitivity to noise and limitations in the number of snapshots [26,27,28,29,30,31].

While traditional DOA estimation methods can deliver satisfactory performance under specific operating conditions, they are plagued by prominent limitations. These drawbacks encompass compromised estimation precision in low-SNR environments, insufficient snapshot numbers, scenarios involving coherent signals, and intricate propagation channels. Furthermore, these approaches are burdened with excessive computational complexity and high sensitivity to parameter configuration factors that severely impede their practical implementation in real-world systems [32,33]. In recent years, deep learning (DL) has emerged as a transformative paradigm in various domains, including computer vision, natural language processing, and speech recognition. Its ability to automatically learn hierarchical feature representations from raw data has spurred interest in applying DL techniques to DOA estimation tasks [34,35,36,37,38]. DL-based methods can effectively capture complex nonlinear relationships within the data, thereby enhancing robustness against noise and multipath effects. For instance, convolutional neural networks (CNNs) have been employed to extract spatial features from array covariance matrices, while recurrent neural networks (RNNs) and long short-term memory (LSTM) networks have been utilized to model temporal dependencies in sequential signal data [39,40,41,42]. Hybrid architectures combining CNNs and LSTMs have also been proposed to leverage both spatial and temporal information for improved DOA estimation accuracy [43,44]. Despite the progress made, existing methods typically overlook the optimization of the covariance matrix, leading neural networks to learn the nonlinear mapping from the complex covariance matrix directly to DOAs. This increases the learning burden on the model and makes it difficult to integrate with existing classical attention mechanisms, thereby limiting the performance of neural networks [39,40,41,42,43,44]. Although the array covariance attention (ACA) mechanism has been proposed to enhance DOA estimation performance in non-ideal noise environments, it overlooks the optimized representation of covariance matrices, thus failing to fully exploit the potential of classical attention mechanisms in DOA estimation [45].

This paper proposes a dual-branch attention-based CNN-LSTM network (DACL-Net) for DOA estimation. The data flows into two parallel computational branches. The first branch is the spatial branch, which optimizes the covariance matrix through a two-dimensional Fourier transform (2D-FT) so that angles appear as peaks in the magnitude spectrum. Spatial features are then extracted via residual blocks. In this process, the coordinate attention (CA) mechanism enhances the spatial–local perception capability, enabling the network to focus more on peak regions [46]. The second branch is the temporal branch. Since time series are more susceptible to noise, we incorporate a spectrum attention mechanism (SAM) for the long short-term memory (LSTM) network [47], improving the noise robustness of the temporal branch. Finally, the outputs of the two branches are fused through a linear layer to produce the DOA estimation result. The main contributions of this paper are summarized as follows:

1. DACL-Net introduces a novel 2D-FT-based input representation that transforms the array covariance matrix into the spatial frequency domain. This transformation effectively converts the original covariance matrix into a dark image with bright spots, where each DOA corresponds to a distinct peak in the magnitude spectrum. By leveraging this representation, the model enables classical computer vision attention mechanisms to focus on these peak regions, thereby improving feature discriminability and DOA estimation accuracy. This 2D-FT preprocessing serves as the cornerstone of our approach, integrating physical prior knowledge into the DL framework and reducing the network’s learning burden.
2. A lightweight adaptive filtering module, the SAM, is employed as a preprocessor for the LSTM branch. SAM adaptively suppresses noise components in the time-domain signals through a learnable frequency-domain mask, while residual connections preserve crucial phase information. This architecture offers a novel paradigm for temporal modeling in array signal processing and can be extended to other related tasks.
3. We propose an improved cross-entropy (CE) loss function, angle-weighted cross entropy (AWCE), that assigns higher weights to training samples corresponding to edge angles based on a sine-based weighting scheme. This mechanism enhances the model’s focus on challenging marginal samples, thereby improving overall estimation consistency across the entire angular range. The weighting strategy is general and can be incorporated into other loss functions beyond the one used in this work.

This paper is structured as follows. Section 2 formulates the uniform linear array (ULA) signal model and analyzes the information embedded within the spatial spectrum. Section 3 provides a detailed description of the proposed DACL-Net architecture. In Section 4, the performance of the proposed framework is evaluated through simulated experiments, where its advantages and limitations are discussed in comparison with existing methods. Section 5 concludes the paper.

## 2. Signal Model

**Figure 1:**
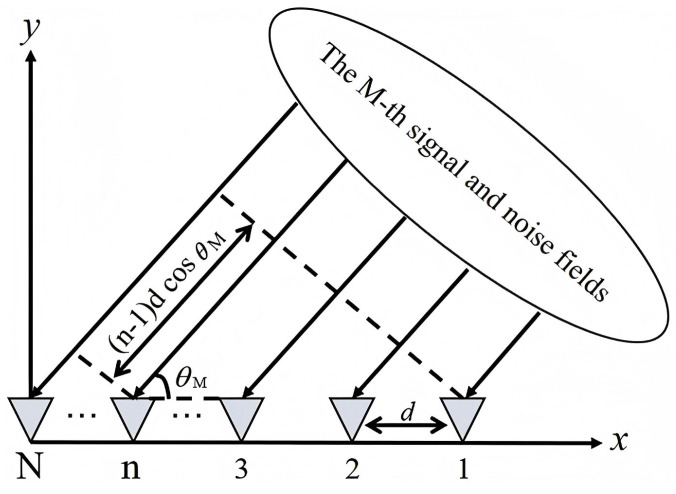
Uniform linear array used in this work.

Consider a ULA composed of *N* sensor elements arranged along a straight line with an inter-element spacing of *d*. Assume that *M* far-field narrowband signals from distinct directions impinge on the array, as depicted in Figure 1. Each signal is assumed to be uncorrelated with the others and additive noise.

Let *T* denote the number of snapshots collected by each array element. The signal received by the *n*-th element at time *t* is provided by the following:

(1) $$x_{n}\left(t\right)=\sum_{i=1}^{M}s_{i}\left(t\right)e^{-j\frac{2\pi }{\lambda }\left(n-1\right)d\sin\theta_{i}}+n_{n}\left(t\right),\;n=1,2,\ldots ,N,$$

where λ=c/f is the wavelength of the signal, with *c* being the speed of light and *f* the signal frequency. The term $s_{i}\left(t\right)$ represents the complex envelope of the *i*-th signal at time *t*, $\theta_{i}$ is the direction of arrival of the *i*-th signal, and $n_{n}\left(t\right)$ is additive white Gaussian noise with zero mean and known variance.

The received signal vector across the array at time *t* can be expressed as follows:

(2) $$x\left(t\right)={\left[x_{1}\left(t\right),x_{2}\left(t\right),\ldots ,x_{N}\left(t\right)\right]}^{T}.$$

This leads to the compact matrix form:

(3) x(t)=A(θ)s(t)+n(t),

where $s\left(t\right)={\left[s_{1}\left(t\right),s_{2}\left(t\right),\ldots ,s_{M}\left(t\right)\right]}^{T}$ is the signal vector, n(t) is the noise vector, and $A\left(\theta \right)=\left[a\left(\theta_{1}\right),a\left(\theta_{2}\right),\ldots ,a\left(\theta_{M}\right)\right]$ is the array manifold matrix. The steering vector $a(\theta_{i})$ for a signal arriving from angle $\theta_{i}$ is defined as follows:

(4) $$a\left(\theta_{i}\right)={\left[1,e^{-j\frac{2\pi }{\lambda }d\sin\theta_{i}},e^{-j\frac{4\pi }{\lambda }d\sin\theta_{i}},\ldots ,e^{-j\frac{2\pi }{\lambda }\left(N-1\right)d\sin\theta_{i}}\right]}^{T}.$$

In practical scenarios, the array covariance matrix $R=E[x\left(t\right)x{\left(t\right)}^{H}]$ is estimated using the sample covariance matrix:

(5) $$\hat{R}=\frac{1}{T}\sum_{t=1}^{T}x\left(t\right)x{\left(t\right)}^{H},$$

where ${\left(\cdot \right)}^{H}$ denotes the conjugate transpose. The spatial spectrum is then constructed by scanning over a predefined angular grid $\Theta =\{\theta_{1},\theta_{2},\ldots ,\theta_{L}\}$. For each candidate angle θ, the steering vector a(θ) is used to compute the beam output amplitude:

(6) $$Z\left(\theta \right)=\left|a{\left(\theta \right)}^{H}\hat{R}a\left(\theta \right)\right|.$$

The resulting spatial spectrum $\left\{Z\left(\theta_{1}\right),Z\left(\theta_{2}\right),\ldots ,Z\left(\theta_{L}\right)\right\}$ exhibits prominent peaks near the true DOAs. This characteristic forms the foundation of subspace-based methods, where the identification of these spectral peaks is essential for accurate direction finding.

To facilitate supervised learning, the ground-truth DOA information is encoded as a binary label vector $Y={\left[y_{1},y_{2},\ldots ,y_{L}\right]}^{T}$:

(7) $$y_{i}=\left\{\begin{array}{cc} 1, & \mathrm{if}\;\theta_{i}\;\mathrm{is}\;a\;\mathrm{true}\;\mathrm{DOA}\\ 0, & \mathrm{otherwise} \end{array}\right..$$

This labeling scheme enables the network to distinguish signal components from noise during training and enhances its ability to accurately localize source directions in the spatial spectrum.

## 3. Proposed Method

This paper presents a DL-based DOA estimation method. The proposed architecture employs a dual-branch design. One branch performs feature optimization via a 2D-FT, followed by residual blocks equipped with a CA module. The other branch consists of an LSTM network and SAM. SAM adaptively filters out noise from the input, and LSTM extracts temporal features. We first introduce each component individually, then describe the integrated architecture composed of these components, and finally present an optimized CE loss function.

### 3.1. Two-Dimensional Fourier Transform

For a ULA receiving *M* far-field signals, the ideal covariance matrix can be expressed as $R=\sum_{i=1}^{M}\sigma_{i}^{2}a\left(\theta_{i}\right)a{\left(\theta_{i}\right)}^{H}+\sigma_{n}^{2}I$, where $a(\theta_{i})$ is the steering vector. Under the far-field narrowband assumption, the steering vector has a Vandermonde structure: $a\left(\theta_{i}\right)={\left[1,e^{-j\frac{2\pi }{\lambda }d\sin\theta_{i}},\ldots ,e^{-j\frac{2\pi }{\lambda }\left(N-1\right)d\sin\theta_{i}}\right]}^{T}$. The 2D-FT of the outer product $a\left(\theta_{i}\right)a{\left(\theta_{i}\right)}^{H}$ essentially computes the following:

(8) $$F_{i}\left(u,v\right)=\sum_{m=0}^{N-1}\sum_{n=0}^{N-1}e^{-j\frac{2\pi }{\lambda }d\left(m\sin\theta_{i}-n\sin\theta_{i}\right)}e^{-j2\pi \left(\frac{um}{N}+\frac{vn}{N}\right)}.$$

This results in energy concentration around the spatial frequencies (u,v) satisfying the following:

(9) $$\frac{u}{N}\approx \frac{d\sin\theta_{i}}{\lambda }\;\mathrm{and}\;\frac{v}{N}\approx -\frac{d\sin\theta_{i}}{\lambda },$$

Forming a localized high-energy region (peak) in the magnitude spectrum |F(u,v)|. Areas without signal sources correspond to noise-only components, which under the white Gaussian noise assumption yield relatively flat and low-magnitude responses, appearing as dark regions.

The mathematical formulation of the 2D-FT applied to the covariance matrix $\hat{R}$ is provided by the following:

(10) $$F\left(u,v\right)=\sum_{m=0}^{M-1}\sum_{n=0}^{N-1}\hat{R}\left(m,n\right)\cdot e^{-j2\pi \left(\frac{um}{M}+\frac{vn}{N}\right)},$$

where $\hat{R}\left(m,n\right)$ denotes the element at the *m*-th row and *n*-th column of the estimated covariance matrix, *M* and *N* represent its dimensions, and u,v are the spatial frequency indices. The magnitude spectrum |F(u,v)| is then computed. In this spectral representation, each DOA corresponds to a localized high-energy region, while areas without signal sources remain relatively dark. This structured representation allows subsequent convolutional layers and attention modules to effectively localize and emphasize the angular information, thereby reducing the learning burden of the network and improving feature discriminability. The tensor formed by concatenating the phase spectrum ∠F(u,v) and the magnitude spectrum |F(u,v)| serves as the input to the spatial branch to preserve complete spatial frequency information.

### 3.2. Coordinate Attention

The CA module is integrated into the residual block to enhance the model’s capability to capture directional features of sound sources. This module decomposes the input feature map into a pair of direction-aware feature vectors by performing pooling operations separately along the two spatial dimensions. These vectors are then encoded into a pair of attention maps, with each map capturing contextual information from one directional perspective of long-range spatial dependencies in the input feature map. Through this decomposition transformation, the module effectively captures remote dependencies in one spatial direction while preserving precise positional information in the other. Finally, the resulting two attention maps are applied to the input feature map to emphasize feature information relevant to the target sound source’s direction, thereby improving the accuracy and robustness of DOA estimation. The core algorithm is outlined in Algorithm 1.
**Algorithm 1** Coordinate Attention (CA)
 **Input:** 
Input feature map $X\in R^{C\times H\times W}$
 **Output:** 
Output feature map $Y\in R^{C\times H\times W}$  1:# Global average pooling along height and width directions$z_{h}=\frac{1}{W}\sum_{j=1}^{W}X\left(:,:,j\right)$    # Height descriptor $z_{h}\in R^{C\times H}$$z_{w}=\frac{1}{H}\sum_{i=1}^{H}X\left(i,:,:\right)$    # Width descriptor $z_{w}\in R^{C\times W}$  2:# Concatenate and transform via shared 1D convolution$z=\mathrm{Concat}(z_{h},z_{w})$    # $z\in R^{C\times (H+W)}$f=δ(Conv1D(z))    # $f\in R^{\frac{C}{r}\times \left(H+W\right)}$  3:# Split and apply sigmoid activation$f_{h},f_{w}=\mathrm{Split}\left(f,\left[H,W\right]\right)$$g_{h}=\sigma \left({\mathrm{Conv}1D}_{h}\left(f_{h}\right)\right)$    # Height attention $g_{h}\in R^{C\times H}$$g_{w}=\sigma \left({\mathrm{Conv}1D}_{w}\left(f_{w}\right)\right)$    # Width attention $g_{w}\in R^{C\times W}$  4:# Apply attention weights to input features$Y\left(i,j,:\right)=X\left(i,j,:\right)\times g_{h}\left(i,:\right)\times g_{w}\left(j,:\right)$

### 3.3. Spectrum Attention Mechanism

Since the original time-domain signals are more affected by noise than the covariance matrix, the SAM module is designed to adaptively remove noise from the input signals in the frequency domain. It operates by transforming the input signal into the frequency domain using the discrete Fourier transform (DFT). A learnable mask is then applied to weight different frequency components, allowing the network to emphasize informative frequencies while suppressing noise. Finally, the inverse DFT (IDFT) is used to convert the filtered frequency-domain representation back into the time domain. The core algorithm is outlined in Algorithm 2.
**Algorithm 2** Spectrum Attention Mechanism (SAM)
 **Input:** 
Captured signal sequence $x^{n}$
 **Output:** 
$x_{sam}^{n}$  1:Initialize: All-ones learnable array $mask^{n}$  2:# Transform the input series into frequency domain$sp^{n}\leftarrow DFT\left(x^{n}\right)$  3:# Element-wise multiply spectrum by mask$masked\_sp^{n}\leftarrow sp^{n}\cdot mask^{n}$  4:# Transform the spectrum back into the time domain$x_{sam}^{n}\leftarrow IDFT\left(masked\_sp^{n}\right)$

Since the SAM operates on the entire frequency domain of the signal, it can lead to the loss of phase information when transformed back to the time domain via IDFT. To mitigate this, we integrate the module into the network using the following residual connection scheme:

(11) $$x_{filtered}^{n}=x^{n}+x_{sam}^{n}.$$

This design preserves the crucial phase information contained in the original input. Furthermore, by allowing the filter to indirectly learn the frequency components of the noise rather than a direct mapping to the clean signal, this skip connection facilitates an easier learning process.

### 3.4. CNN-LSTM

The CNN-LSTM hybrid architecture can be structured in either serial or parallel configurations. Our model employs a parallel design to integrate spatial and temporal features, fusing these complementary representations to enhance DOA estimation accuracy. Below we provide separate introductions to the CNN and LSTM components.

#### 3.4.1. Convolutional Neural Network

**Figure 2:**
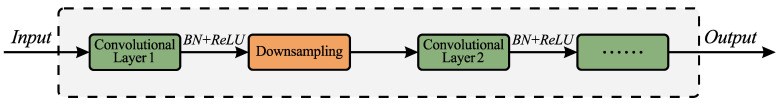
Basic structure of convolutional blocks.

In its fundamental form, a CNN comprises stacked convolutional layers that progressively extract hierarchical features from input data. As illustrated in Figure 2, these layers are often interleaved with downsampling operations to enhance computational efficiency and expand receptive fields. In our architecture, the conventional convolutional stack is replaced by residual blocks to facilitate deeper network design while maintaining training stability.

(1) Convolutional layer

The feature extraction operation using convolutional kernels is mathematically expressed as follows:

(12) $$\begin{array}{cc} Z^{l+1}\left(i,j\right) & =\left[Z^{l}⊗w^{l+1}\right]\left(i,j\right)+b\\ \; & =\sum_{k=1}^{K_{l}}\sum_{x=1}^{f}\sum_{y=1}^{f}\left[Z_{k}^{l}\left(s_{0}i+x,s_{0}j+y\right)w_{k}^{l+1}\left(x,y\right)\right]\\ \; & +b\left(i,j\right)\in \left\{0,1,\ldots ,L_{l+1}\right\},\\ L_{l+1} & =\frac{L_{l}+2p-f}{s_{0}}+1, \end{array}$$

where $Z^{l}$ and $Z^{l+1}$ denote the input and output of the (l+1)-th layer, respectively. *K* indicates the channel count, Z(i,j) represents the pixel value at position (i,j), *b* denotes the bias term, $w^{l+1}$ corresponds to the convolutional kernel weights at layer l+1, $L_{l+1}$ specifies the output size, $s_{0}$ and *f* indicate the stride and kernel size, respectively, and *p* refers to the padding size.

(2) Residual block

To overcome the limitations of plain CNNs in deep architectures, we employ residual blocks that incorporate skip connections. These connections enable direct feature propagation across layers, alleviating gradient vanishing and enabling the construction of deeper networks. The core operation of a residual block is formulated as follows:

(13) $$y=F(x,\left\{W_{i}\right\})+x,$$

where x and y are the input and output vectors of the block, and $F(x,\left\{W_{i}\right\})$ represents the residual mapping to be learned. In our design, the residual path consists of convolutional layers and CA modules, which enhance the spatial feature discriminability crucial for DOA estimation. Downsampling is achieved by utilizing convolutional layers with a stride of 2 within these residual blocks, eliminating the need for separate pooling layers.

#### 3.4.2. Long Short-Term Memory Network

**Figure 3:**
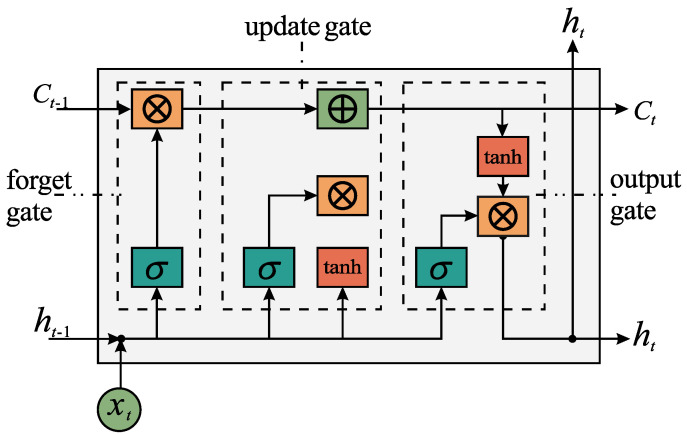
The structure of LSTM.

Recurrent neural networks have evolved through numerous architectural innovations, with LSTM representing one of the most significant developments. The LSTM unit incorporates four key components: a forget gate $f_{t}$ with parameters $\left\{W_{xf},W_{hf},W_{cf},b_{f}\right\}$, an update gate $i_{t}$ with parameters $\left\{W_{xi},W_{hi},W_{ci},b_{i}\right\}$, an output gate $o_{t}$ with parameters $\left\{W_{xo},W_{ho},W_{co},b_{o}\right\}$, and a candidate state component $g_{t}$ with parameters $\left\{W_{xc},W_{hc},W_{cc},b_{c}\right\}$. The architectural diagram appears in Figure 3.

Let $x_{t}$, $c_{t}$, and $h_{t}$ represent the input, cell state, and hidden state of the current timestep, while $h_{t-1}$ and $c_{t-1}$ denote the hidden state and cell state from the previous timestep.

The computational procedures for the gates and states at time *t* are formulated as follows:

(14) $$\left\{\begin{array}{cc} \; & f_{t}=\sigma \left(W_{xf}\cdot x_{t}+W_{hf}\cdot h_{t-1}+W_{cf}\cdot c_{t-1}+b_{f}\right)\\ \; & i_{t}=\sigma \left(W_{xi}\cdot x_{t}+W_{hi}\cdot h_{t-1}+W_{ci}\cdot c_{t-1}+b_{i}\right)\\ \; & g_{t}=\tanh\left(W_{xc}\cdot x_{t}+W_{hc}\cdot h_{t-1}+W_{cc}\cdot c_{t-1}+b_{c}\right)\\ \; & c_{t}=i_{t}g_{t}+f_{t}c_{t-1}\\ \; & o_{t}=\sigma \left(W_{xo}\cdot x_{t}+W_{ho}\cdot h_{t-1}+W_{co}\cdot c_{t-1}+b_{o}\right)\\ \; & h_{t}=o_{t}\tanh\left(c_{t}\right) \end{array}.\right.$$

In DOA estimation, the signals received by the sensor array not only exhibit spatial correlation but also demonstrate temporal correlation. LSTM, through its gating mechanism, can effectively capture this temporal dependency, thereby enhancing estimation stability under low SNR conditions.

### 3.5. Integrated Architecture

**Figure 4:**
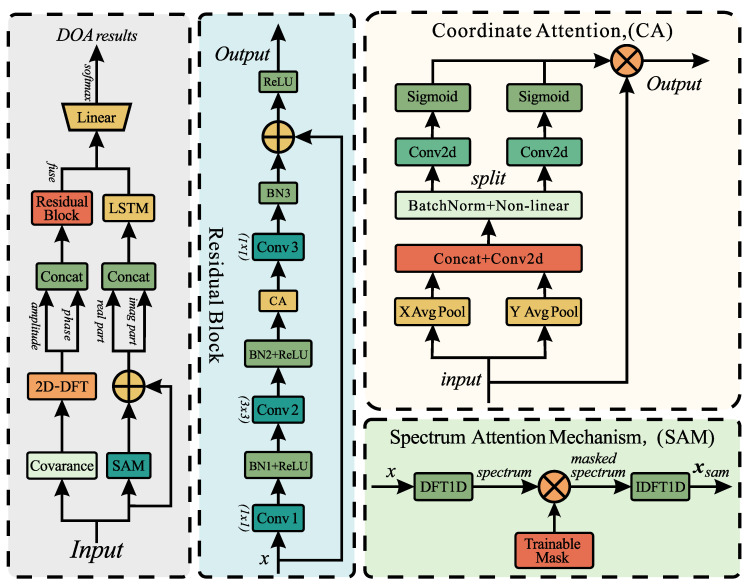
Our proposed integrated architecture.

By integrating the aforementioned components in the manner illustrated in Figure 4, our overall architecture is constructed. Specifically, the input data flows into two parallel branches. The first branch uses a 2D-FT to refine the input covariance matrix. The magnitude and phase information of refined data are then processed through residual blocks embedded with CA modules. The second branch extracts temporal features via an LSTM based on SAM. The outputs of these two branches are then fused by linear layers to produce the final DOA estimation results.

The described fusion structure is named DACL-Net, which offers the following advantages:

(1) DACL-Net is built upon a feature transformation, namely the two-dimensional Fourier transform, which converts cross-correlation information into spatial power distribution. Angles manifest as peaks in the spatial power distribution, resembling bright spots in an image. This enables classical attention mechanisms from the image domain to be effectively utilized. Essentially, this integrates physical prior knowledge from array signal processing into the neural network, significantly reducing the training burden and improving estimation accuracy.
(2) Existing DL-based DOA estimators typically consider only spatial correlation features while neglecting temporal sequence characteristics. DACL-Net, based on a spatio-temporal feature extraction baseline model, integrates dual-branch features, thereby enhancing the robustness and accuracy of DOA estimation.
(3) Although DACL-Net incorporates attention mechanisms and a dual-branch architecture, its overall parameter count remains within a reasonable range. Specifically, the SAM module includes only learnable mask parameters; the CA module achieves lightweight attention computation via one-dimensional convolutions; and the convolutional layers within the residual blocks all employ small-sized kernels. Compared with existing deep CNN and Transformer-based models, DACL-Net maintains high accuracy while exhibiting lower computational complexity and memory usage, making it more suitable for deployment on real-time processing platforms.

### 3.6. Loss Function

In classification tasks, the CE loss is typically employed, and its gradient computation is more straightforward. Compared to the mean squared error (MSE) loss commonly used in regression, it is generally easier to optimize during training and often leads to faster convergence. In the classification task of DOA estimation, angles are discretized into finite categories. The core challenge is that the fitting accuracy of the model for edge angle samples is significantly lower than that for middle angles due to the degradation of array manifold characteristics, reduced SNR, and other issues. To address the problem, this paper designs an AWCE loss function, which enhances the model’s learning attention to edge angle samples by assigning adaptive weights to different angle categories.

#### 3.6.1. Basic Cross Entropy Loss

The standard cross entropy loss for classification tasks is defined as follows:

(15) $$L_{CE}=-\frac{1}{N}\sum_{i=1}^{N}\sum_{c=1}^{C}y_{i,c}\log\left(p_{i,c}\right),$$

where *N* is the number of samples in a batch, *C* is the total number of discrete angle categories, $y_{i,c}\in \left\{0,1\right\}$ is the one-hot encoded ground-truth label of sample *i* corresponding to category *c*, and $p_{i,c}$ is the probability predicted by the model that sample *i* belongs to category *c*. The standard cross-entropy loss assigns equal weights to all angle categories and cannot specifically improve the fitting effect of edge angles, so an angle-dependent weighting mechanism needs to be introduced.

#### 3.6.2. Design of the Angle Weighting Mechanism

Considering the angular characteristics of the ULA, this paper proposes that the weight function is proportional to the absolute value of the sine of the angle, with the following specific form:

(16) w(θ)=1+a·|sinθ|,

where θ is the ground-truth DOA angle of the sample, and a≥0 is the weight adjustment coefficient. The core characteristics of this weighting mechanism are as follows: (1) when θ=0° (middle angle), |sinθ|=0, and the weight w(θ)=1, which is consistent with the weight of the standard cross entropy loss. (2) When θ→±90° (edge angles), |sinθ|→1, and the weight w(θ)=1+a, which amplifies the loss contribution of edge samples and makes the model prioritize learning the features of such samples. (3) The adjustment coefficient *a* can flexibly control the degree of weight enhancement for edge angles: a larger *a* leads to a more significant weight difference between edge angles and middle angles, and the model has a higher fitting priority for edge samples.

For the discrete angle category *c*, its corresponding angle value is $\theta_{c}$, so the weight of category *c* can be expressed as $w_{c}=1+a\cdot \left|\sin\theta_{c}\right|$, and the weight matrix $W\in R^{C}$ is composed of $w_{c}$ corresponding to all categories.

#### 3.6.3. Angle-Weighted Cross Entropy Loss Function

Combining the above weighting mechanism, the final loss function is defined as follows:

(17) $$L_{AWCE}=-\frac{1}{N}\sum_{i=1}^{N}\sum_{c=1}^{C}w_{c}\cdot y_{i,c}\log\left(p_{i,c}\right).$$

To avoid the scale change in loss values introduced by weights, normalization processing can be applied to the weights as follows:

(18) $${\tilde{w}}_{c}=\frac{w_{c}}{\frac{1}{C}\sum_{c=1}^{C}w_{c}},\;L_{AWCE}=-\frac{1}{N}\sum_{i=1}^{N}\sum_{c=1}^{C}{\tilde{w}}_{c}\cdot y_{i,c}\log\left(p_{i,c}\right).$$

The normalized weights ensure that the overall scale of the loss function is consistent with the standard cross entropy loss, while retaining the weighting effect of edge angles.

## 4. Simulation Results

### 4.1. Dataset Generation

In our experiments, acoustic vector sensors (AVS) are employed as the sensing method, and the ULA model described in Section 2 is adopted for dataset acquisition. Specifically, the signal type is a single-frequency signal, and the noise is set to additive white Gaussian noise. The sound velocity *v* is set to 1500 m/s, and the wavelength λ is 1 m. The array configuration consists of 10 sensors with an inter-element spacing of $\frac{\lambda }{2}$. Under these conditions, two sources impinge on the ULA with random angular separations of 1°, 2°, 3°, 4°, or 5°, covering a DOA range of [−90°,90°], thereby establishing an extremely close-spaced scenario. To enhance data diversity, the SNR is varied from −20 dB to 20 dB in 5 dB increments. The number of snapshots is T=500. We generated a complete dataset comprising 18,000 samples, covering various angular separations and noise levels. For model parameter updates, the Adam optimizer is employed with an initial learning rate of 0.0001. The batch size is set to 1800, and the training process is conducted over 1000 epochs. The program is implemented using PyTorch 2.1.2 and executed on a hardware platform equipped with an Intel(R) Core(TM) i9-14900K CPU @ 3.20 GHz and an NVIDIA GeForce RTX 4090 GPU.

### 4.2. Performance of DOA Estimation Model

**Figure 5:**
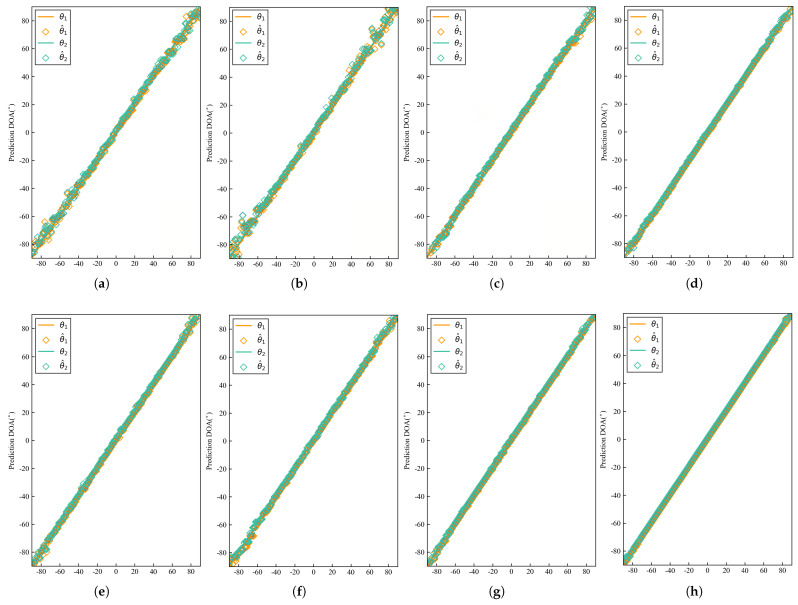
DOA estimation performance when the DOA is set to $\theta_{1},\theta_{2}\in \left[-90{}^{\circ},90{}^{\circ}\right]$ with SNR being 0 dB and the number of snapshots being T = 500. (**a**) DOA estimate of MUSIC. (**b**) DOA estimate of ESPRIT. (**c**) DOA estimate of IMLSE. (**d**) DOA estimate of ILSSE. (**e**) DOA estimate of DCNN. (**f**) DOA estimate of Res-CNN. (**g**) DOA estimate of CNN-LSTM. (**h**) DOA estimate of DACL-Net.

Based on the aforementioned data-generation method, the test set was constructed in the same manner. Far-field narrowband independent signal samples with an identical SNR of 0 dB and an angular separation of 1° were selected from the test set. The DOA estimates for each test sample were computed using both existing algorithms and our proposed method [22,24,39,42,44,48]. To ensure a fair and comprehensive comparison, all DL baseline models were trained and optimized under identical experimental conditions. This included the same dataset split, the same optimizer (Adam) with an identical initial learning rate, and the same number of training epochs, so that each model could achieve its best possible performance. For classical algorithms such as MUSIC and ESPRIT, we adopted widely recognized standard implementations with optimal parameter settings. For MUSIC, the true number of signal sources was provided, and the eigenvalue decomposition method was employed. For methods requiring angular search, we used a fine grid that matched the classification resolution of the neural network to ensure comparable angular resolution across all approaches. The simulation results are presented in Figure 5a–h. The solid line indicates the actual DOA, while the estimated DOA is illustrated by the colored blocks.

**Figure 6:**
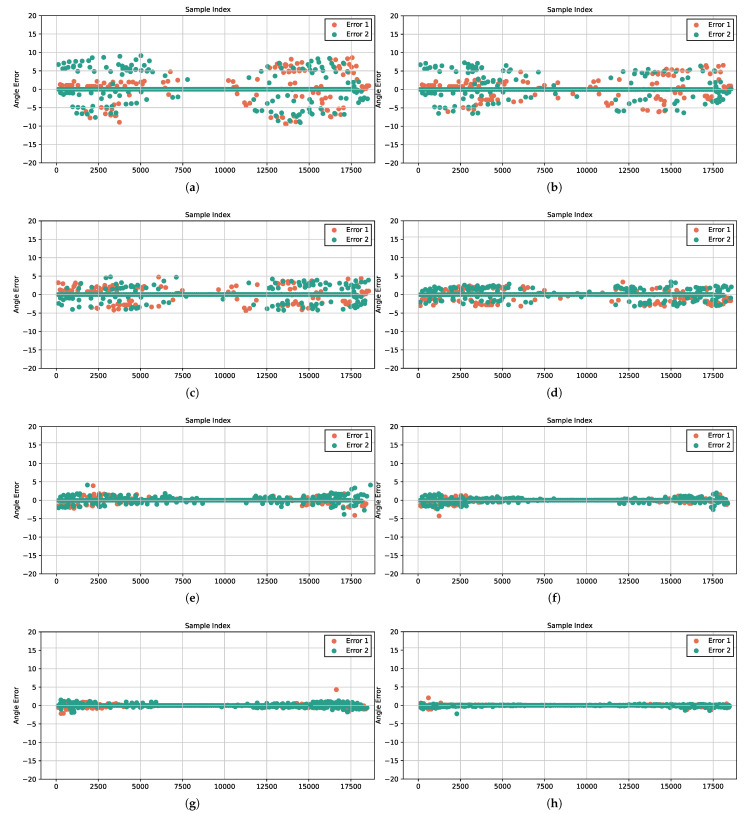
DOA estimation performance when the DOA is set to $\theta_{1},\theta_{2}\in \left[-90{}^{\circ},90{}^{\circ}\right]$ with SNR being 0 dB and the number of snapshots being T = 500. (**a**) DOA error of MUSIC. (**b**) DOA error of ESPRIT. (**c**) DOA error of IMLSE. (**d**) DOA error of ILSSE. (**e**) DOA error of DCNN. (**f**) DOA error of Res-CNN. (**g**) DOA error of CNN-LSTM. (**h**) DOA error of DACL-Net.

As shown in Figure 5a,b, conventional algorithms such as MUSIC and ESPRIT perform poorly at 0 dB SNR, exhibiting highly unstable DOA results, especially near the grid boundaries. It can be observed from Figure 5c,d that the iterative-based algorithms IMLSE and ILSSE achieve improved performance compared to conventional methods, owing to their iterative optimization strategies that enhance robustness against noise and improve estimation stability under low SNR conditions. Figure 5e–h demonstrates that DL-based algorithms achieve the best prediction performance, benefiting from their powerful end-to-end feature learning capability to extract discriminative spatial-spectral features directly from the data. In comparison, our proposed DACL-Net yields predictions closest to the ideal line, achieving high estimation accuracy for both central and edge samples. This balanced performance can be attributed to the proposed AWCE loss function. By assigning higher weights to the training losses of edge-angle samples based on the sine weighting mechanism, the AWCE loss effectively addresses the inherent difficulty in classifying marginal angles due to degraded array manifold characteristics. This weighting strategy ensures that the model allocates sufficient learning capacity to these challenging cases, thereby improving overall estimation consistency across the entire angular range. Additionally, Figure 6a–h displays the prediction errors of each method on the test set samples in the form of scatter plots. These results more clearly demonstrate the superior performance of DACL-Net in low SNR environments. At 0 dB SNR, DACL-Net achieves an RMSE below 0.04°, outperforming other models.

### 4.3. Statistical Performance Analysis

To verify the statistical performance of each algorithm, the root mean square error (RMSE) is expressed as follows:

(19) $$\mathrm{RMSE}=\sqrt{\frac{1}{KM}\sum_{k=1}^{K}\sum_{m=1}^{M}{\left({\hat{\theta }}_{m,k}-\theta_{m}\right)}^{2}},$$

where *K* denotes the number of Monte Carlo trials, *M* represents the number of signal sources, ${\hat{\theta }}_{m,k}$ is the estimated DOA of the *m*-th source in the *k*-th trial, and $\theta_{m}$ is the corresponding ground-truth direction.

#### 4.3.1. Impact of Signal-to-Noise Ratio on Root Mean Square Error

In this experiment, we systematically evaluate the robustness of various algorithms under different SNR conditions. The SNR values for all test samples range from −20 dB to 20 dB with a 5 dB increment, resulting in nine distinct SNR scenarios. To comprehensively assess the estimation error performance under different snapshot conditions, we conduct separate experiments with fixed snapshot numbers of 50, 100, 200, and 500.

**Figure 7:**
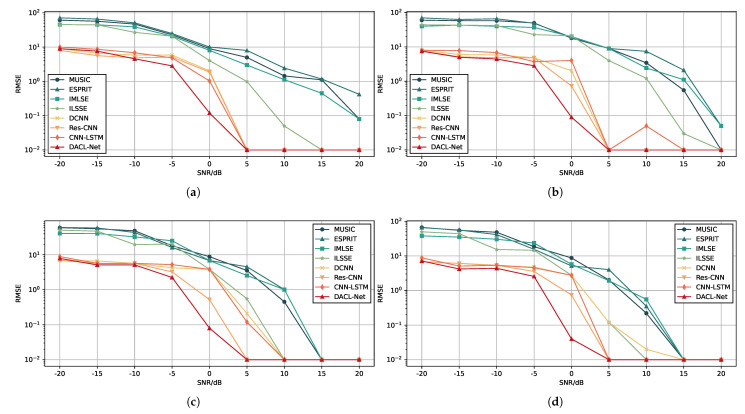
RMSE of DOA estimates. (**a**) Snapshots T=50. (**b**) Snapshots T=100. (**c**) Snapshots T=200. (**d**) Snapshots T=500.

As illustrated in Figure 7a–d, all methods exhibit decreasing RMSE trends as the number of snapshots increases. However, our proposed DACL-Net demonstrates more significant improvement, which can be attributed to its LSTM architecture and the accompanying SAM that effectively leverages abundant temporal information. Table 1 shows the specific data in Figure 7d. Notably, under challenging low-SNR conditions (SNR = −5 dB), DACL-Net maintains superior performance with about 1° RMSE values. This enhanced robustness stems from the integrated adaptive noise filtering module and the attention mechanism that strengthens spatio-temporal feature extraction, enabling more reliable DOA estimation in adverse signal environments.

The superior performance of DACL-Net, and learning-based methods in general, over classical algorithms such as MUSIC and ESPRIT under low SNR conditions can be explained by several key mechanisms. First, classical subspace-based methods rely on accurate estimation of the signal and noise subspaces through eigenvalue decomposition of the sample covariance matrix. In low SNR regimes, the noise subspace becomes dominant, and its orthogonality to the signal subspace is compromised, leading to degraded spectral peaks and increased estimation errors. In contrast, DACL-Net does not depend on explicit subspace decomposition. Instead, it learns a direct mapping from the input data to DOA estimates through hierarchical feature extraction. The 2D-FT preprocessing step transforms the covariance matrix into a spatial frequency representation where signal directions manifest as localized energy peaks, effectively enhancing the SNR in the feature domain. Additionally, the SAM module acts as an adaptive frequency-domain filter, suppressing noise components in the temporal branch, while the CA module in the spatial branch focuses attention on relevant peak regions. This data-driven approach allows the network to capture complex, nonlinear relationships between the received signals and source directions, which are often obscured by noise in classical methods. Furthermore, the model is trained on a diverse dataset encompassing a wide range of SNRs and angular configurations, enabling it to generalize to challenging low-SNR scenarios that are problematic for traditional algorithms. Therefore, DACL-Net’s ability to integrate spatial and temporal features, coupled with attention-guided noise suppression, provides a principled explanation for its robustness in low SNR environments. However, at an SNR of −20 dB, the accuracy of the proposed model deteriorates to a level similar to that of the standard CNN-LSTM baseline.

#### 4.3.2. Impact of Signal-to-Noise Ratio on Estimation Accuracy

**Figure 8:**
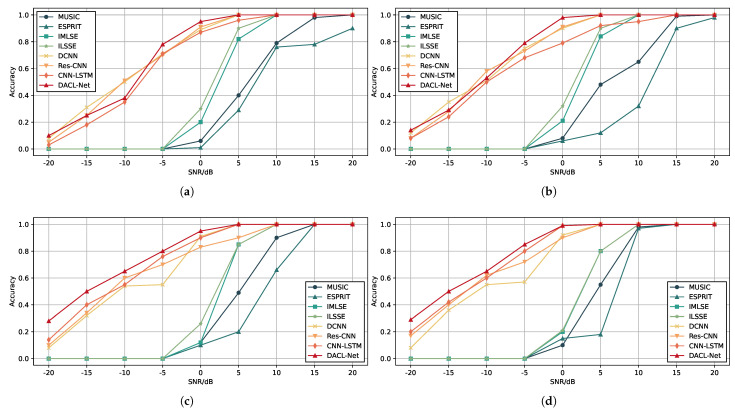
Accuracy of DOA estimates. (**a**) Snapshots T=50. (**b**) Snapshots T=100. (**c**) Snapshots T=200. (**d**) Snapshots T=500.

The experimental parameters in this investigation remain consistent with the previous experiment, while a new evaluation metric is adopted. We define a prediction as correct only when both $\theta_{1}$ and $\theta_{2}$ are accurately estimated. The estimation accuracy is calculated as the proportion of correctly predicted samples within the test set. Figure 8a–d presents the estimation accuracy of DACL-Net compared with other benchmark algorithms. The results demonstrate that our method achieves superior performance under low SNR conditions, attaining the highest angular classification accuracy among all evaluated approaches. Table 2 shows the specific data in Figure 8d. Notably, DACL-Net achieves an accuracy of over 95% at an SNR of 0 dB.

### 4.4. Ablation Study

To verify the effectiveness of each core component in the proposed DACL-Net, we conduct systematic ablation experiments. The baseline model is a standard CNN-LSTM hybrid network without SAM, 2D-FT optimization, a CA module, or AWCE loss. Four ablation variants are designed by sequentially removing individual components, and all models are trained and tested under the same experimental settings (SNR range: −20 dB to 20 dB, snapshots T=500, angular separation: 1°–5°). The RMSE and estimation accuracy at key SNR points (−10 dB, 0 dB, 10 dB) are adopted as evaluation metrics to quantify the contribution of each component.

#### 4.4.1. Ablation Variants Definition

1. DACL-Net (Full model): Integrated with SAM, 2D-FT, CA module, and AWCE loss.
2. Variant 1 (w/o SAM): Removed the SAM.
3. Variant 2 (w/o 2D-FT): Removed the 2D-FT optimization.
4. Variant 3 (w/o CA): Retained 2D-FT but removed the CA module.
5. Variant 4 (w/ CE Loss): Replaced AWCE loss with standard CE loss.

#### 4.4.2. Ablation Experimental Results

The performance of all ablation variants is summarized in Table 3 and Table 4. All values are averaged over 10 Monte Carlo trials to ensure statistical reliability.

Compared to the full model, Variant 1 (w/o SAM) shows a 49.4% RMSE increase and a 31.4% accuracy decrease at −10 dB SNR. This confirms that the adaptive noise filtering capability of SAM effectively suppresses noise interference in low SNR environments, laying a foundation for subsequent feature extraction. Variant 2 (w/o 2D-FT) exhibits the most significant performance degradation among all variants, with RMSE increased by approximately 110.8% and accuracy decreased by approximately 45.8% at −10 dB SNR. This underscores the pivotal role of the 2D-FT input transformation in optimizing spatial feature representation and forming the dark image with bright spots that enable effective attention mechanism operation. Variant 3 (w/o CA) performs worse than the full model, with RMSE increased by 8.5% and accuracy decreased by 7.2% at −10 dB SNR. This verifies that the CA module further refines the spatially optimized features from 2D-FT, improving the ability to capture directional information of target sources and enhance peak localization. Variant 4 (w/ CE Loss) has higher RMSE and lower accuracy than the full model, with RMSE increased by 40.0% and accuracy decreased by 25.7% at −10 dB SNR. This demonstrates that the sine-based weighting mechanism of AWCE loss effectively enhances the attention to edge-angle samples, alleviating the problem of edge sample misclassification caused by array manifold degradation.

In conclusion, all core components of DACL-Net play important roles in improving DOA estimation performance. The 2D-FT transformation proves to be the most critical component for feature optimization, while SAM provides essential noise robustness in challenging environments. The CA module and AWCE loss further refine spatial feature extraction and training efficiency.

### 4.5. Computational Efficiency Evaluation

**Figure 9:**
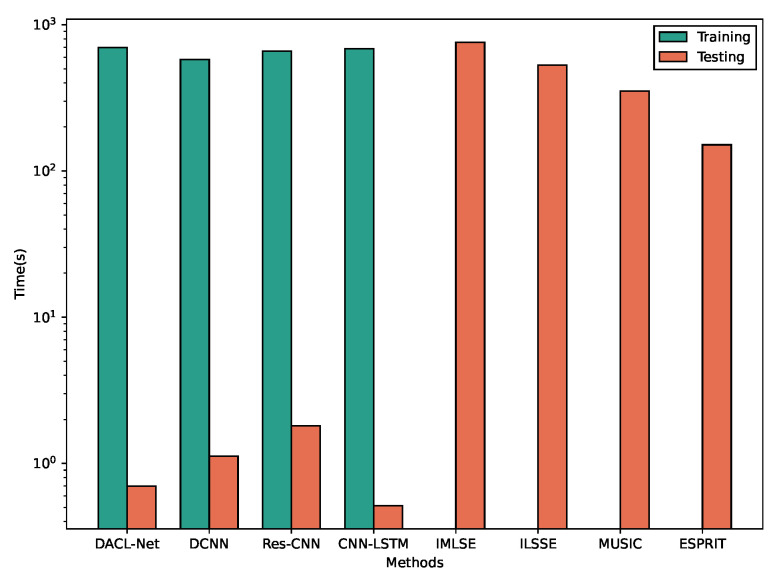
Execution time comparison for various methods.

To evaluate the computational efficiency of the proposed DACL-Net, we additionally constructed a test set containing 10,000 samples with a fixed snapshot number of 500, employing varying angles and SNR levels. All randomly generated samples were processed using different methods. The evaluation is conducted on the same hardware platform described in Section 4.1, ensuring consistency across all methods. The total prediction time is recorded. The results are summarized in Figure 9 and Table 5.

Compared to the other three DL-based methods, DACL-Net requires longer training time due to its more complex architecture. This is mainly attributed to its LSTM branch, which takes raw long-sequence signals as direct input. While traditional methods bypass the training phase altogether, experimental results confirm that well-trained DL models can achieve efficient DOA estimation even with moderate computational resources. Although DACL-Net’s structural complexity leads to slightly longer inference time compared to the original CNN-LSTM, it still maintains substantially faster computation than DCNN and Res-CNN. This enables our method to deliver both real-time performance and high measurement accuracy.

### 4.6. Generalization Ability with Multiple Sources

To evaluate the generalization capability of DACL-Net in scenarios with more than two sources, we conducted additional experiments involving three, four, and five far-field uncorrelated narrowband signals. The array configuration remains the same as described in Section 4.1, with N=10 sensors and inter-element spacing d=λ/2. The DOAs of the sources are randomly generated within [−90°,90°] with a minimum angular separation of 1° to simulate closely spaced sources. Three representative SNR levels are considered: −10 dB, 0 dB, and 10 dB. The number of snapshots is fixed at T=500. For each source count, 2000 test samples are generated.

The performance is evaluated using estimation accuracy, where a prediction is considered correct only if all source DOAs are correctly estimated. The results are compared with two representative DL baselines: Res-CNN and CNN-LSTM. The results are summarized in Table 6.

The results demonstrate that DACL-Net consistently outperforms both Res-CNN and CNN-LSTM across all source counts and SNR levels, especially under low SNR conditions. As the number of sources increases, all methods exhibit performance degradation due to increased spatial interference and higher model complexity. However, DACL-Net shows better robustness, with a smaller drop in accuracy compared to the baselines. This indicates that the proposed dual-branch architecture with attention mechanisms effectively extracts and fuses spatio-temporal features even in more challenging multi-source scenarios.

### 4.7. Physical Interpretability

A key advantage of DACL-Net lies in its enhanced physical interpretability compared to purely data-driven deep learning approaches for DOA estimation. By incorporating the 2D-FT preprocessing step, the model explicitly leverages the known structure of the array covariance matrix under the far-field narrowband assumption. This transformation maps the original complex-valued correlation data into a spatial frequency domain representation where signal directions manifest as distinct spectral peaks, analogous to bright spots on a dark image. This representation is not arbitrary. It directly corresponds to the spatial Fourier transform of the array manifold, a well-established concept in array signal processing. Consequently, the subsequent CNN and attention modules operate on a physically meaningful feature space, allowing the network to focus on regions of high spatial energy corresponding to potential source directions. This design choice bridges the gap between classical signal processing theory and deep learning, providing a clearer pathway to understand how the network arrives at its estimates. Furthermore, the SAM module’s frequency-domain masking operation can be interpreted as an adaptive noise suppressor, learning to attenuate frequency bins dominated by noise while preserving signal components. This imbues the temporal branch with a degree of interpretability regarding its noise robustness.

### 4.8. Limitations

Despite the strong performance of DACL-Net, several limitations warrant consideration. First, the current model design is based on a ULA geometry and a far-field narrowband signal model. Its performance under near-field conditions, with broadband signals, or on arbitrary array geometries has yet to be verified. Second, although its computational cost is lower than that of many iterative classical methods, it remains higher than some DL baselines during training. This is primarily due to the dual-branch structure and additional attention modules. For application scenarios with strict constraints on training time or device learning capabilities, this could pose a challenge. Third, the current evaluation of the method is based on simulation experiments. The model’s performance on measured data requires further validation. Overcoming these bottlenecks is crucial for advancing DACL-Net toward robust and general-purpose DOA estimation in practical systems.

## 5. Conclusions

This paper presents DACL-Net, a dual-branch attention-based CNN-LSTM network for DOA estimation. The spatial branch employs a 2D-FT to optimize the covariance matrix, causing angular information to appear as peaks in the magnitude of the spatial frequency spectrum. This representation allows the attention mechanisms, commonly used in computer vision, to effectively guide the neural network towards these peaks, thereby enhancing feature discriminability and improving DOA estimation accuracy. The SAM serves as an adaptive filter in the temporal branch, effectively mitigating the impact of noise on time-series signals. The deep features extracted from the two branches are fused through a linear layer to output the final DOA estimation results. Experimental results demonstrate the superior performance of DACL-Net, especially in low SNR environments.

This work primarily focuses on the ULA under far-field narrowband assumptions. Extending the proposed framework to more general array geometries, such as uniform circular arrays (UCAs) or non-uniform arrays, and adapting it to near-field or wideband scenarios, present important directions for future research. The current performance evaluation is based on simulations. Thus, validation with real-world measured data is a crucial next step to assess the model’s practicality and robustness. 

## Figures and Tables

**Table 1:** RMSE of DOA estimates given 500 snapshots, with SNR varying from −20 dB to 20 dB.

Methods	−20 dB	−15 dB	−10 dB	−5 dB	0 dB	5 dB	10 dB	15 dB	20 dB
MUSIC	66.79	55.08	48.37	18.55	8.74	2.01	0.22	0.01	0.01
ESPRIT	70.46	56.98	50.66	15.51	5.14	4.01	0.35	0.01	0.01
IMLSE	38.01	35.08	30.37	23.55	5.74	1.92	0.55	0.01	0.01
ILSSE	49.80	44.43	15.92	14.17	2.74	0.12	0.01	0.01	0.01
DCNN	6.79	6.08	5.17	4.75	0.24	0.12	0.02	0.01	0.01
Res-CNN	6.90	5.99	5.37	3.55	0.27	0.01	0.01	0.01	0.01
CNN-LSTM	5.79	5.08	5.22	3.12	0.18	0.01	0.01	0.01	0.01
**DACL-Net**	**5.99**	**2.42**	**1.55**	**0.98**	**0.04**	**0.01**	**0.01**	**0.01**	**0.01**

**Table 2:** Accuracy of DOA estimates given 500 snapshots, with SNR varying from −20 dB to 20 dB.

Methods	−20 dB	−15 dB	−10 dB	−5 dB	0 dB	5 dB	10 dB	15 dB	20 dB
MUSIC	0%	0%	0%	0%	10%	55%	98%	100%	100%
ESPRIT	0%	0%	0%	0%	15%	18%	97%	100%	100%
IMLSE	0%	0%	0%	0%	20%	80%	100%	100%	100%
ILSSE	0%	0%	0%	0%	21%	80%	100%	100%	100%
DCNN	8%	36%	55%	57%	92%	100%	100%	100%	100%
Res-CNN	17%	40%	62%	72%	90%	100%	100%	100%	100%
CNN-LSTM	20%	42%	60%	80%	99%	100%	100%	100%	100%
**DACL-Net**	**22%**	**50%**	**65%**	**85%**	**99%**	**100%**	**100%**	**100%**	**100%**

**Table 3:** RMSE comparison of ablation variants. (Unit: °).

Model Variant	SNR = −10 dB	SNR = 0 dB	SNR = 10 dB
DACL-Net (Full Model)	4.37 ± 0.06	0.04 ± 0.03	0.01 ± 0.01
Variant 1 (w/o SAM)	6.53 ± 0.09	0.15 ± 0.05	0.01 ± 0.01
Variant 2 (w/o 2D-FT)	9.21 ± 0.11	0.10 ± 0.06	0.02 ± 0.02
Variant 3 (w/o CA)	4.74 ± 0.08	0.04 ± 0.06	0.02 ± 0.02
Variant 4 (w/ CE Loss)	6.12 ± 0.07	0.13 ± 0.03	0.05 ± 0.04

**Table 4:** Estimation accuracy comparison of ablation variants. (Unit: %).

Model Variant	SNR = −10 dB	SNR = 0 dB	SNR = 10 dB
DACL-Net (Full Model)	65.3 ± 1.2	97.0 ± 0.2	99.9 ± 0.1
Variant 1 (w/o SAM)	44.8 ± 1.5	85.2 ± 0.4	99.9 ± 0.1
Variant 2 (w/o 2D-FT)	35.4 ± 1.8	90.6 ± 0.5	99.1 ± 0.7
Variant 3 (w/o CA)	60.6 ± 1.4	96.1 ± 0.5	98.2 ± 0.4
Variant 4 (w/ CE Loss)	48.5 ± 1.3	88.4 ± 0.8	95.8 ± 0.8

**Table 5:** Time consumption comparison across different DOA estimation methods.

Time(s)	DACL-Net	DCNN	Res-CNN	CNN-LSTM	IMLSE	ILSSE	MUSIC	ESPRIT
**Training**	698.1286	578.3883	660.3481	685.4442	/	/	/	/
**Testing**	0.7006	1.1223	1.8123	0.5144	758.3347	529.6822	352.6552	151.0608

**Table 6:** Estimation accuracy with multiple sources under different SNR conditions.

Method	3 Sources			4 Sources			5 Sources		
	−10 dB	0 dB	10 dB	−10 dB	0 dB	10 dB	−10 dB	0 dB	10 dB
Res-CNN	28.5%	85.2%	99.8%	15.1%	70.3%	98.5%	8.7%	52.4%	95.2%
CNN-LSTM	35.2%	88.6%	99.9%	18.9%	75.8%	99.1%	12.3%	60.5%	96.8%
**DACL-Net**	**42.6%**	**94.5%**	**99.9%**	**25.4%**	**82.7%**	**99.4%**	**16.8%**	**68.9%**	**97.5%**

## Data Availability

Relevant information and codes are available from the corresponding author if required.
